# Developing a new individual earthquake resilience questionnaire: A reliability and validity test

**DOI:** 10.1371/journal.pone.0245662

**Published:** 2021-01-22

**Authors:** Ning Jiang, Jingxia Cheng, Zhihong Ni, Yansheng Ye, Rujun Hu, Xiaolian Jiang

**Affiliations:** 1 Institute for Disaster Management and Reconstruction, Sichuan University, Chengdu, Sichuan, China; 2 West China Hospital/West China School of Nursing, Sichuan University, Chengdu, Sichuan, China; Charles Sturt University - Port Macquarie Campus, AUSTRALIA

## Abstract

Earthquakes pose serious threats to the world. Good individual resilience can cope with disaster well, but there were few appropriate assessment tools. The purpose of this study was to develop a new individual earthquake resilience questionnaire and test its reliability and validity. First, we built the framework of the individual earthquake resilience questionnaire based on expert interviews. Then, we established the initial version of questionnaire and used the Delphi method and item selection to modify it by qualitative and quantitative methods. Finally, we built the final version of questionnaire (contained 4 dimensions and 17 items) and tested the reliability and validity. The Cronbach’s α values of the four dimensions were between 0.79 and 0.91, the split-half reliabilities were between 0.85 and 0.93, and the test-retest reliabilities were between 0.72 and 0.80. The item content validity indexes were between 0.87–1, and the average questionnaire content validity index was 0.94. The correlation coefficients between each item and dimension with the total questionnaire ranged from 0.79–0.90 and 0.66–0.79, respectively. We used exploratory factor analysis to identify four common factors with a cumulative variance contribution rate of 74.97%. The questionnaire is a valid and reliable tool to measure individual resilience in the context of earthquake disasters.

## Introduction

Earthquakes are the most destructive disaster in the world, resulting in a large number of casualties and very large economic losses, seriously restricting the development of human society. Resilience refers to the capability of a system to be restored to its original state after disturbance [1]. Individual resilience is the ability of a person to cope with traumatic events, including maintaining a relatively stable, healthy mental condition and physical function; gaining experience; and maintaining positive emotions [2]. Individual resilience also refers to individuals’ good function in all respects during or after exposure to risk factors [3]. In the field of disaster science, individual resilience is defined as the ability of people to adapt well psychologically, emotionally and physically in the face of disasters, threats and challenges without causing permanent damage to themselves, their interpersonal relationships and their development ability [4]. Overall, individual resilience indicates the ability of individuals to resist, absorb, recover or adapt after being attacked. In this paper, we defined individual resilience as the ability of a person to withstand and adapt well and recover as soon as possible when suffering adverse events.

For the evaluation of individual resilience, Friborg et al. developed the Resilience scale for Adults (RSA), which consists of 45 items covering 5 dimensions: personal competence, social competence, family coherence, social support and personal structure [5]. The 25-item Connor-Davidson resilience scale (CD-RISG-25) was developed by Connor and Davidson. The scale included ability, instinct, acceptance of change, control and mental influence 5 dimensions and 25 items. Each rated on a 5-point scale, with higher scores reflecting greater resilience [6]. Wei and Taormina built the multidimensional measure of personal resilience which contained determination, endurance, adaptability, recuperability 4 dimensions and 40 items with a 5-point scale [7]. Campbell and Stein believed that the 5-dimension structure of CD-RISG-25 was unstable, so they revised the scale and formed a single-dimensional CD-RISG-10 [8]. On the basis of the CD-RISG-25 and RSA, Slepian et al. developed a Pain Resilience Scale, an assessment tool suitable for the psychological resilience of pain patients, included Cognitive/Affective Positivity and Behavioral Perseverance 2 dimensions and 14 items [9]. Almahmoud examined the multidimensional resilience of adults with blood transfusion-dependent thalassemia, including their psychological adjustment, treatment adherence, social functioning and occupational functioning [10]. The Child and Youth Resilience Measure (CYRM) was a common tool used to measure four aspects of resilience, including the individual, relationships, the community and culture [11]. Crann and Barata suggested that resilience was experienced as multiple cognitive, emotional, and behavioral shifts across three thematic areas: toward resistance, in the experience of control, and toward positivity [12]. Barger et al. developed a tool to measure resilience using a model of the 7Cs of resilience, including competence, confidence, character, connection, coping, contribution, and control [13]. Hjemdal O developed the Resilience Questionnaire for Adolescents (READ), which contains five factors: personal competence, social competence, structured style, family cohesion and social resources [14]. Although individual resilience assessment tools were gradually increasing, most of them focused on psychological resilience, adolescents or patients resilience. Scales of earthquake disasters resilience for the residents were relatively rare. Earthquakes cause huge loses to the world. Individual earthquake resilience means personal abilities to respond and adapt to disasters, which could develop self-protection and survival skills during disasters. So evaluating the earthquake disaster resilience, finding out the influencing factors, and then enhancing the residents’ capacities of adaptation and response are important issues that researches need to pay attention to. Disaster nurses play important roles in disaster prevention, relief and postdisaster reconstruction. Our essential task is to maintain individual health in disasters. Therefore, it is urgent to develop a credible individual resilience evaluation tool based on the earthquake disaster from a professional perspective. In this study we developed a new individual resilience questionnaire which contained 4 dimensions (health status, mental resilience, social adaptation and disaster response capability) and 17 items. By evaluating the effectiveness of the questionnaire, we provided a reliable basis for the researches of earthquake disaster resilience.

## Material and methods

First, we built the framework of individual resilience questionnaire based on expert interviews. Then, we created the item pool. We used the Delphi method to construct the individual resilience questionnaire and weight the items. Finally, we conducted an investigation in Dujiangyan, a city that was affected by the Wenchuan earthquake, to select items and test the reliability and validity.

### Expert interviews

A semi structured interview approach was used in the expert interviews. Objective sampling was used to select 10 experts. The inclusion criteria for the experts were as follows: 1) work or research in the area of community nursing, disaster nursing or disaster resilience for more than 10 years; 2) still perform this work or research at the time of the study; and 3) have associate professor title or graduate degree or higher.

### Delphi method

Objective sampling was used to select 24 experts on community nursing, disaster nursing or disaster prevention and mitigation for the Delphi method. The inclusion criteria were the same as those of the expert interviews.

Three Delphi rounds were conducted in this study: the first and second rounds were modifying and improving the questionnaire, and the third round was determining the index weights. The expert positive coefficient, authority coefficient (Cr) and coordination coefficient were used for evaluation.

Criteria for indicator selection. The items were scored based on their importance by the experts and selected through the critical value method, which involves the selection of the items with scores above the critical values of the arithmetic mean (AM), full mark rate (FMR) and coefficient of variation (CV). We calculated the critical value of the AM as the mean minus the standard deviation. The critical value of the FMR was determined in the same way. Items scores that were higher than the threshold were selected. The critical value of the CV was equal to the mean plus the standard deviation. Item scores that were lower than the threshold were selected. The revisions suggested by the experts were also fully considered.

### Item selection

A cross-sectional study was carried out among 110 urban residents living in Dujiangyan, a city that was affected by the Wenchuan earthquake. The inclusion criteria for the residents were as follows: 1) live in the community for more than half a year; 2) at least 15 years old; 3) could complete questionnaires independently or with the help of investigators; and 4) informed consent.

SPSS19.0 software was used to analyze the data. Frequency distribution method, discrete trend method, and correlation coefficient method were used to select items. Items that meet the norms in 2 or more methods will be deleted. The criteria as follows [15, 16]: 1) response rate <10% with 3 or more grades in the frequency distribution method; 2) standard deviation (SD) less than 0.75 in the discrete trend method; 3) correlation coefficient with the total questionnaire or its dimensions were less than 0.4.

### Reliability and validity test

A cross-sectional study was carried out among urban residents living in Dujiangyan city. And the inclusion criteria were the same as those for the item selection subjects.

This study covered the main city areas of Dujiangyan using a multistage sampling strategy. First, all subdistricts under the city were taken as the first-level sampling unit. Second, a stratified sampling method was then used to extract 12 communities from subdistricts at a rate of 25%. Finally, a sample map describing the residential locations in the selected communities was drawn, on which all households were numbered. We used systematic sampling to select households at a rate of 1.5%. Residents over fifteen years old in each household participated in the investigation.

Analyses were performed using SPSS 19.0. Validity was assessed through the content validity, internal structure correlation and exploratory factor analysis (EFA) [17]. The reliability was obtained through the Cronbach’s alpha, split-half reliability and test-retest reliability.

### Ethics aspects

The study protocol was approved by the Human Subjects Ethics Sub-committee of Sichuan University (registration number: 2017–386). All participants agreed to participate in the study and informed consents obtained from adolescents were written and adults were verbal. Consents were obtained from a parent or guardian on behalf of any participants under the age of 18. The ethics committee approved this procedure.

## Results

### Expert interviews

10 experts were interviewed and their basic information was shown in Table 1.

**Table 1:** Basic information of interview experts (n = 10).

Code	Gender	Age	Education	Years of working/research	Specialty
A	F	52	Doctor	30	Disaster nursing
B	F	45	Undergraduate	19	Community nursing
C	F	34	Doctor	10	Disaster resilience
D	M	36	Doctor	11	Disaster resilience
E	F	54	Doctor	36	Disaster nursing
F	F	44	Undergraduate	20	Community nursing
G	F	52	Doctor	34	Disaster nursing
H	F	56	Graduate	36	Community nursing
I	F	39	Graduate	13	Disaster nursing
J	F	45	Graduate	19	Community nursing

We analyzed the materials collected in experts interviews and identified four themes: health status, mental resilience, social adaptation and disaster response capacity. Parts of experts’ discussion were extracted after themes.

#### Theme 1: Health status

In any situation, the focus of nursing is human health. Residents in good health can adapt to changes and recover quickly when experiencing an earthquake, which plays an important role in community resilience.

Expert B commented on health status, stating “Residents having good physical quality, for example young adults being the major group is good for resilience. When an earthquake occurs, residents have enough physical strength to save themselves, help each other and reduce the disability rate.” Expert F said, “If the physical condition of community residents is good, it may be beneficial to the post-disaster reconstruction.”

#### Theme 2: Mental resilience

People should adapt to setbacks and be able to recover from a traumatic event. In addition to adaptation and the restoration of physical conditions, individual resilience is also concerned about mental status. Tenacity involves people being adaptable and resilient to adversity, which are vital to individual resilience.

Expert E pointed out, “People who have experienced large earthquakes, especially seen their loved ones seriously injured or killed in the disaster, may have psychological trauma, which will affect their subsequent recovery.” Expert C said, “Earthquakes may cause physical injuries to residents. some people may be disabled which might be a great blow to their spirits. If these people have strong mental resilience, they will recover better in the future. But if they remain depressed, their recovery will be slow.” And Expert H said, “People with good mental resilience tend to be optimistic, flexible and have better adaptability and coping ability. When earthquake occurs, they can cope with adversity better, which can improve the resilience of communities.”

#### Theme 3: Social adaptation

Individual resilience is derived from interactions between multiple dimensions, including the individual, family, school, work unit and community. Individual resilience is dynamic and associative as a result of the interaction between people and external factors, of which social adaptation is an important part. Social adaptation includes role adaptation, problem adaptation, environmental adaptation and interpersonal adaptation.

Expert D commented on social adaptation, stating “After the earthquake, the surrounding environment of residents may change a lot, and a variety of problems may arise. If you have good adaptability, these problems may be solved easily.” Expert I said, “Adaptation is important for community reconstruction after a disaster. The earthquake may change your social role, such as family, work or study environment change, so you will face a completely new environment. Social adaptation also is conducive to post-disaster reconstruction.”

#### Theme 4: Disaster response capacity

Only people who possess the capacity to take action before, during and after disasters can adapt to changes and recover quickly. Prevention before an earthquake is beneficial to alleviate the loss caused by seismic disasters. Coping with disasters, including administering medical aid to myself and others, as well as being equipped with basic survival skills and knowledge about disease prevention after earthquakes, can significantly enhance individual resilience.

Expert A pointed out, “Earthquake response capacity is important. If you don’t have basic self-save skills, you can not safely evacuate in an earthquake. And if you get hurt, what you can do is waiting for someone to rescue, then you are in a very passive situation.” And Expert J said, “What do you do when an earthquake occurs, where to hide, and what to pay attention to. These skills you need to master. If you are lucky enough to get out, what do you do if someone gets hurt or buried under the collapsed house? I’m sure that if we have sufficient disaster response capacity, there will be fewer casualties.”

### Building individual resilience questionnaire-initial version

Based on the expert interviews and the consultation of the literature, we created the individual resilience questionnaire-initial version including 4 dimensions and 15 items (Table 2). In health status, the physical health of residents can greatly affect the residents’ ability to prevent, resist and recover from disasters [18]. Individual physical health is mainly reflected by physical condition, behavior and thinking [18]. First, if individuals physical condition are better than that of their peers, these residents can evacuate to safe areas faster and they also have enough strength to join the reconstruction team in the recovery stage [19]. The second is behavior, namely the self-care ability. People with strong self-care ability can handle their own affairs well, and they can give more aid to other people when they need help [20]. Third, thinking, namely cognitive response ability. Good cognitive competence can help people accurately identify the extent of damage and quickly find living space when the surrounding environment changes dramatically [21]. In mental resilience, Taylor et al. believed that good mental resilience can be reflected in the ability to maintain a stable mental level and generate positive emotions when dealing with emergencies [22, 23]. Tact, self-discipline, curiosity, optimism, resilience, connection, adaptability, emotional control, patience et al. are all traits of mental resilience. But in the context of earthquakes, emotion control, optimism, resilience and restorability are particularly important [22–25]. In social adaptation, social adaptability can be understood from four levels: sensory adaptation, behavioral adaptation, cognitive adaptation and personality adaptation from the perspective of cognitive psychological process [26]. And in socialization, social adaptation includes adaptation to social living environment, social role and social activities. In this study, earthquakes may lead to a series of problems, so individuals should be aware of their own abilities and give play to their advantages to deal with problems properly. At the same time, the earthquake disaster may cause changes of original lives, and individuals need to be competent to this changed condition [27]. Finally, individuals who want to better adapt to society need to have close relatives and friends and keep in touch with them regularly. Such individuals with good interpersonal relationships can get help from outside in time when they are in trouble. In disaster response capacity, the Chinese government has decreed the “National Disaster Prevention and Mitigation Plan (2016–2020)”, which clearly sets out the goal of “improving the popularization rate of earthquake prevention and control knowledge”. This study was based on the earthquake disaster, assessing ability mainly included the estimation ability, escape ability, survival skills, and primary rescue ability. When the earthquake occurs, individuals should be alert to the earthquake, understand the threat may face, and judge secondary disaster risk [28]. Earthquakes have strong destructive powers on buildings and surrounding environments. How to evacuate and escape quickly from danger is a very important response ability. Generally speaking, individuals should know how to escape or avoid danger indoors and outdoors and self-rescue when trapped [29]. Individuals also need basic survival skills, such as access to clean water, food and fuel. When they live in shelters, they need to pay attention to safe water, disease prevention and hygienic diet. Casualties may occur after earthquakes, individuals should have the ability to rescue buried people, hemostasis, bandaging, fixation, cardiopulmonary resuscitation, transport and other primary rescue capabilities [30].

**Table 2:** Individual resilience questionnaire-initial version.

Dimension	Item
Health status	I am generally in good health.
	I have the ability to perform the activities of daily living, including eating food, dressing, bathing, going to the toilet, etc.
	I have good cognition, with an IQ above the average level.
Mental resilience	I always have a positive view when suffering difficulties.
	I can recover from setbacks quickly.
	I try to solve a problem instead of giving up easily when suffering difficulties.
	I believe I can control my emotions when an earthquake strikes.
Social adaptation	I can play my roles in daily life well.
	I am fully aware of my abilities and can cope with problems well.
	I can adapt quickly when the environment changes.
	I can get along with others well.
Disaster response capacity	I have a strong awareness of earthquakes and can preliminarily assess earthquake disasters. ^a^
	I know exactly how to escape and evacuate when an earthquake strikes. ^b^
	I have basic survival skills needed after an earthquake. ^c^
	I can administer first aid, as well as medical aid to myself and others, when an earthquake strikes. ^d^

^a^ I am aware of potential earthquake threats, can perceive earthquake warning signs, and accurately assess the grade of an earthquake and the risk of secondary disasters.

^b^ I know how to escape or avoid danger indoors and outdoors and self-rescue when trapped.

^c^ I have skills including getting water, food, and fuel and keeping water and food clean.

^d^ I have skills including searching for and rescuing those who are buried, performing hemostasis, applying bandages, administering CPR and carrying the wounded.

### Delphi method

A total of 22 experts participated in the first round and 19 in the second (Table 3). The first and second round recovery rates were 91.67% and 86.36%, respectively which showed expert positive coefficient was good. The Cr of the first round was 0.87 and second round was 0.86 displaying good expert authority coefficient. The expert coordination coefficient (Kendall’s W) of the first round was 0.20 (P<0.001), and that of the second round was 0.39 (P<0.001) (S1 Table).

**Table 3:** Basic information of Delphi experts.

Content	Categories	First round (n = 22)		Second round (n = 19)	
		Number of people	Percentage	Number of people	Percentage
Gender	M	7	31.82%	5	26.32%
	F	15	68.18%	14	73.68%
Age	30~	7	31.82%	7	36.84%
	40~	11	50.00%	8	42.11%
	50~	3	13.64%	3	15.79%
	60~	1	4.54%	1	5.26%
Education	Undergraduate	1	4.54%	1	5.26%
	Graduate	3	13.64%	3	15.79%
	Doctor	18	81.82%	15	78.95%
Professional title	Medium-grade	3	13.64%	3	15.78%
	Associate Professor	9	40.91%	8	42.11%
	Professor	10	45.45%	8	42.11%
Specialty	Disaster nursing	8	36.36%	7	36.84%
	Community nursing	7	31.82%	7	36.84%
	Disaster prevention and mitigation	7	31.82%	5	26.32%
Years of working/research	10~	12	54.54%	10	52.63%
	20~	5	22.73%	4	21.05%
	30~	5	22.73%	5	26.32%

### Modification of the items

#### First round

The results of the first round were analyzed according to predefined criteria. The AM was 4.43±0.25, and the threshold value was 4.18. The FMR was 54.24±14.83, and the threshold was 29.41. The CV was 15.65±5.08, and the threshold value was 21.73 (S1 Table). No items were deleted according to the criteria. Some suggestions for amending or adding items from the experts were as follows.

Eighteen percent of experts suggested amending “I have good cognition, with an IQ above the average level” to “I can think clearly and communicate with the outside world.” The experts suggested that because it is impractical to measure IQ, we should instead pay more attention to the ability to think and communicate. Thirteen percent of experts thought that excellent physical performance could help people escape to reduce casualties when an earthquake occurs. Thus, we added an item regarding physical activities: “I can move freely and easily.” Fourteen percent of experts believed that tenacity is one of the typical characteristics of mental resilience in addition to self-control, positivity and a capacity for recovery. Consequently, the following item was added: “I believe that difficulties make me stronger.” Nine percent of experts advised adding an item about social resources that are beneficial to postdisaster reconstruction; the following item was added: “I am good at finding and using social resources (staff, funds, supplies, skills, social relations and so on).” Nine percent of experts advised simplifying the items “I will try to solve a problem instead of giving up easily when suffering difficulties” and “I am fully aware of my abilities and can cope with problems well” as follows: “I do not give up easily when suffering difficulties” and “I can cope with problems well,” respectively. Twenty-three percent of experts advised that the items in the “disaster response capacity” dimension be written in the first person. Fourteen percent of experts suggested listing simpler search and rescue capabilities because the initial capabilities listed were too advanced for the average respondent. Therefore, we changed these items as follows: “I am aware of potential earthquake threats, can perceive earthquake warning signs, and preliminarily assess the grade of an earthquake and the risk of secondary disaster” and “I know the principles of searching for and rescuing those who are buried and have preliminary skills including performing hemostasis, applying bandages, administering CPR and carrying the wounded.”

#### Second round

In the second round, the AM was 4.48±0.16, and the threshold value was 4.32. The FMR was 53.22±12.75, and the threshold was 40.47. The CV was 13.08±2.27, and the threshold value was 15.35 (S1 Table). No items were deleted in the second round according to the criteria. The suggestions provided by the experts were as follows.

According to 5% of the experts, the item “I have the ability to perform the activities of daily living, including eating, dressing, bathing, going to the toilet, etc.” was similar to “I can move freely and easily,” so they suggested deleting the former.

Five percent of the experts thought the items “I can think clearly and communicate with the outside world” and “I can play my roles in daily life well” were beyond the comprehension of some people with low education levels. Thus, we amended them as follows: “I can think clearly and communicate with others” and “I can play my roles in daily life well, such as studying hard as a student, doing my own job as a worker, taking care of my family as a parent, etc.”

The individual resilience questionnaire was finalized after two Delphi rounds; it contains 4 dimensions (health status, mental resilience, social adaptation and disaster response capability) and 17 items.

#### Third round

The purpose of third round was index weighting. The analytic hierarchy process was used to calculate the weight of each dimension. The weight of each item was determined as the percentage of each score in relation to the sum of all items in the same dimension.

Nineteen questionnaires were sent to experts, 16 of which were returned with a judgment matrix of the index weights. The composite reliability of one expert’s judgment matrix was greater than 0.1, which did not indicate relative consistency, so the matrix was excluded. The weights are shown in Table 4.

**Table 4:** Weights of the individual earthquake resilience questionnaire.

Dimension	Weight	Item	Weight
Health status	0.307	C1 I am generally in good health.	0.331
		C2 I can move freely and easily.	0.342
		C3 I can think clearly and communicate with others.	0.327
Mental resilience	0.254	C4 I believe I can control my emotions when an earthquake strikes.	0.193
		C5 I always have a positive view when suffering difficulties.	0.199
		C6 I do not give up easily when suffering difficulties.	0.206
		C7 I can recover from setbacks quickly.	0.212
		C8 I believe that difficulties make me stronger.	0.190
Social adaptation	0.175	C9 I can play my roles in daily life well, such as studying hard as a student, doing my own job well as a worker, taking care of my family as a parent, etc.	0.198
		C10 I can cope with problems well.	0.205
		C11 I can adapt quickly when the environment changes.	0.207
		C12 I can get along with others well.	0.183
		C13 I am good at finding and using social resource (staff, funds, supplies, skills, social relations and so on).	0.207
Disaster response capacity	0.264	C14 I have basic earthquake disaster assessment ability. ^a^	0.231
		C15 I know how to escape after shocks. ^b^	0.260
		C16 I have basic survival skills needed after an earthquake. ^c^	0.260
		C17 I can administer first aid. ^d^	0.249

^a^ I am aware of potential earthquake threats, can perceive earthquake warning signs, and preliminarily assess the grade of an earthquake and the risk of secondary disaster.

^b^ I know how to escape or avoid danger indoors and outdoors and self-rescue when trapped.

^c^ I have skills including getting water, food, and fuel and keeping water and food clean.

^d^ I know the principles of searching for and rescuing those who are buried and have preliminary skills of first aid including performing hemostasis, applying bandages, administering CPR and carrying the wounded.

### Item selection

The frequency of answer options in 17 items was summarized, and there were no items with response rate <10% in 3 or more grades. The SD of item C3 and C12 were less than 0.75. The correlation coefficient of 17 items with the total questionnaire and its dimensions were greater than 0.4. (Table 5) To sum up, no item in the questionnaire was deleted according to the item selection principle. The individual resilience questionnaire—final version contained 4 dimensions (health status, mental resilience, social adaptation and disaster response capability) and 17 items (S2 Table).

**Table 5:** Item discrete trend and correlation coefficient.

Item	Item discrete trend		Correlation coefficient				
	Mean	SD	Total questionnaire	Dimension	Dimension	Dimension	Dimension
				1	2	3	4
C1	4.04	0.95	0.64	0.86			
C2	4.48	0.76	0.76	0.90			
C3	4.50	0.71	0.72	0.86			
C4	4.16	0.84	0.78		0.87		
C5	4.19	0.80	0.78		0.92		
C6	4.15	0.79	0.82		0.92		
C7	4.19	0.76	0.81		0.93		
C8	4.15	0.81	0.72		0.84		
C9	4.27	0.80	0.85			0.88	
C10	4.24	0.80	0.82			0.90	
C11	4.14	0.80	0.80			0.88	
C12	4.33	0.73	0.76			0.82	
C13	3.91	0.91	0.79			0.87	
C14	3.65	1.00	0.76				0.89
C15	3.68	0.96	0.78				0.94
C16	3.65	0.98	0.81				0.93
C17	3.36	1.09	0.74				0.91

### Reliability and validity test

#### Basic information of residents

A total of 1048 questionnaires were sent to residents, and 952 were returned, for a response rate of 90.84%. Among the surveyed residents, males accounted for 40.38%, and females accounted for 59.62%; the proportion of people aged 30–60 years was relatively high. Due to the privacy of personal income, only 714 people provided annual disposable income data (Table 6).

**Table 6:** Basic information of residents.

Content		Number of residents (n)	Constituent
			percentage (%)
Gender	Men	384	40.38
	Women	568	59.62
Age	Under 18	25	2.65
	18~	122	12.82
	30~	184	19.28
	40~	165	17.37
	50~	199	20.87
	60~	136	14.30
	70~	121	12.71
Education	Primary school and below	159	16.69
	Junior high school	243	25.52
	Senior high school	247	25.97
	Graduate and above	303	31.82
Employment	Yes	410	43.09
	No	185	19.46
	Retirement	298	31.28
	Student	59	6.17
Annual disposable income (10,000 RMB)	Less than 2	90	12.61
	2~	296	41.46
	4~	181	25.35
	6~	69	9.66
	8~	78	10.92

### Reliability tests

#### Cronbach’s alpha

The Cronbach’s alphas for the health status, mental resilience, social adaptation and disaster response capability dimensions were.79,.91,.89 and.90, respectively, which were considered acceptable.

#### Split-half reliability

The split-half reliabilities of the four factors were 0.85, 0.93, 0.90 and 0.90, respectively, which were considered acceptable (Table 7).

**Table 7:** Split-half reliability (n = 952).

Dimension	Number of items	Split-half coefficient	Split-half reliability
Health status	3	0.74**	0.85
Mental resilience	5	0.88**	0.93
Social adaptation	5	0.82**	0.90
Disaster response capacity	4	0.83**	0.90

**P<0.01.

#### Test-retest reliability

Thirty participants participated in a retest of the individual resilience evaluation questionnaire after 2 weeks. We calculated the correlations for a type I error of.05. The rest-retest reliabilities of the health status, mental resilience, social adaptation and disaster response capability were statistically significant, with values of 0.80, 0.72, 0.79 and 0.80, respectively.

### Validity tests

#### Content validity

The item content validity index (I-CVI) ranged from 0.87–1, and the scale average content validity index (S-CVI/Ave) was 0.94, indicating good content validity.

#### Relative validity of the items and total questionnaire

The correlations of the 17 items with the dimensions ranged from 0.81–0.89, and the dimensions with the total questionnaire ranged from 0.79–0.90. All dimensions and items were positively and significantly correlated with the total questionnaire (P<0.01) (Table 8).

**Table 8:** Correlations between the items and the dimensions (n = 952).

Item	Dimension			
	Health status	Mental resilience	Social adaptation	Disaster response capacity
1	0.83**			
2	0.88**			
3	0.83**			
4		0.81**		
5		0.89**		
6		0.88**		
7		0.88**		
8		0.85**		
9			0.84**	
10			0.88**	
11			0.85**	
12			0.82**	
13			0.81**	
14				0.86**
15				0.89**
16				0.89**
17				0.87**
Total questionnaire	0.79**	0.88**	0.90**	0.83**

**P<0.01.

#### Exploratory factor analysis

The Kaiser-Meyer-Olkin value was 0.95, which was considered acceptable. The 17 items were divided among the four factors, and the cumulative variance contribution rate was 74.97% using the principal component analysis method. All items were finally accepted, as they loaded significantly onto the four components with loading factors greater than 0.5 (Table 9).

**Table 9:** Factor loadings after rotation.

Item	Factor loading value			
	1	2	3	4
1	.37	.19	.14	.64
2	.15	.19	.25	.85
3	.09	.28	.27	.79
4	.28	.71	.11	.37
5	.21	.77	.33	.24
6	.19	.73	.41	.20
7	.28	.74	.36	.17
8	.26	.63	.48	.15
9	.20	.45	.64	.28
10	.24	.41	.71	.27
11	.30	.36	.71	.14
12	.20	.25	.74	.27
13	.54	.18	.56	.23
14	.81	.17	.21	.12
15	.81	.26	.19	.20
16	.81	.26	.20	.17
17	.82	.15	.17	.11

## Discussion

In this paper, for the first time, we built a framework for an individual earthquake resilience questionnaire through expert interviews. Then we created the initial version of questionnaire conducted three Delphi rounds to improve and weight the items and dimensions according to the experts’ ratings and suggestions. Finally, we tested the reliability and validity of the questionnaire through actual measurement. The development process of the questionnaire was scientific and reliable.

### Reliability tests

The Cronbach’s alpha reveals the internal consistency validity of a questionnaire, with values between 0 and 1. The greater the value is, the higher the reliability of the questionnaire [31]. In this study, the Cronbach’s alphas of the questionnaire and each dimension were between 0.79–0.94, which showed that the questionnaire and each dimension had good internal consistency.

Split-half reliabilities also indicate the internal reliability, with a value ≥0.7 [32]. Similar to Cronbach’s alpha, the higher the split half reliability is, the higher the degree of internal consistency. In this study, the split half reliability values of the questionnaire and each dimension were greater than 0.8, indicating that the internal consistency was good.

Test-retest reliability is commonly used to determine external reliability, which mainly tests the consistency of a questionnaire at different stages with a value ≥0.7. In this study, a correlation analysis was conducted with the results at two-week intervals, and the results were all greater than 0.7, indicating that the questionnaire had good stability over time.

### Validity tests

Content validity is used to evaluate whether each item of the questionnaire reflects the actual content of the variables to be measured. The I-CVI should be no less than 0.78, and the S-CVI/Ave should be no less than 0.9 [33]. In this study, the I-CVI was higher than 0.85, and the S-CVI/Ave was 0.94, which was greater than 0.9, indicating that the content validity of the questionnaire was good.

A correlation analysis mainly indicates the internal consistency of a questionnaire with correlation coefficients greater than 0.3 [34]. In this study the correlation coefficients between the dimensions, items and the total questionnaire were all greater than 0.6, indicating that the internal consistency of the questionnaire was good.

In this study, we conducted an EFA, which is a powerful multivariable analysis examining the structure and relationship between variables, as well as the construct validity of a questionnaire [35]. The cumulative variance contribution rate indicates the extent to which the common factors explain the total variance. Factor loadings indicated the correlations between an index and a common factor. In this study, 4 common factors basically represented the overall structure of the questionnaire, and the loading values of each item on the corresponding factors were all above 0.5, indicating that the questionnaire had good structural validity.

## Conclusions

This study firstly built a framework for the questionnaire through expert interviews. Then developing the initial version of questionnaire under Delphi methods and testing the effectiveness through empirical study. The entire process of this study was scientific and the questionnaire was a valid and reliable tool to measure individual resilience in earthquakes. We can use this questionnaire to assess the earthquake disaster resilience of community residents. The questionnaire can be used to identify the residents with low disaster resilience in the community, which can lay a foundation for the follow-up intervention of improving the resilience residents, so as to improve the whole community resilience. The individual resilience questionnaire was mainly developed and verified in the context of Chinese culture. If the tool is used in other countries with different cultural backgrounds, its effectiveness should be further tested.

## Supporting information

S1 File Expert interview guide—English version and Chinese version. (DOCX)

S2 File Adolescent written informed consent—English version and Chinese version. (DOCX)

S1 Table Expert positive coefficient, expert authority coefficient and expert coordination coefficient in Delphi method. (DOCX)

S2 Table Individual resilience questionnaire based on earthquake disaster from the perspective of nursing—English version and Chinese version. (DOCX)

S1 Data Original data of individual resilience. (XLSX)
