# Supplementary material for: Developing a new individual earthquake resilience questionnaire: A reliability and validity test
Source: PLoS One. 2021 Jan 22;16(1):e0245662. doi: 10.1371/journal.pone.0245662 (PMC7822309; doi:10.1371/journal.pone.0245662)
Supplement: S1 File — (DOCX) [file pone.0245662.s001.docx]

**Construction of individual resilience questionnaire based on earthquake disaster from the perspective of nursing:**

**Expert interview guide**

Hello, I am a doctoral candidate in the Institute for Disaster Management and Reconstruction, Sichuan University, majoring in disaster Nursing. Earthquakes posed huge threats to many parts of the world. With urbanization accelerating, cities are more vulnerable to massive earthquakes. As an important part for community, residents not only directly experience the earthquake disaster, but also play an irreplaceable role in pre-disaster prevention and post-disaster reconstruction. The resilience theory emphasizes the system's good adaptation to adverse environment and pays attention to the protective factors and their interaction mechanism in the adaptation process, which is significant to disaster prevention and conrol. The main purpose of this interview is to construct an individual resilience questionnaire based on earthquake disaster from the perspective of nursing, which could provide a reference for reducing disaster losses and promoting post-disaster reconstruction. Now it is the construction stage of the questionnaire. I need you to answer a few questions to help me build the framework, which will take about 30 minutes. If you have any questions or discomfort during the conversation, please let me know immediately. There is no limit if you want to interrupt or withdraw from this conversation. Our conversation will be recorded with a recording pen, and the content is used only in this study. I guarantee that the data will be kept absolutely confidential and your identity will not be disclosed in any step. Are you ready? Shall we start now?

1. What impact do you think will the earthquake have on the community?

2. What impact will the earthquake have on the residents living in communities?

3. Do you know about resilience? If so, where do you learn about resilience?

4. What is individual resilience?

5. From the perspective of nursing, what aspects do you think individual resilience includes?

6. As for the earthquake disaster, which aspects you mentioned before should be paid special attention to? And what are the reasons? Please elaborate on it.

**护理视角下基于地震灾害的个体韧性评价体系构建**

**访谈提纲**

导语：您好，我是四川大学灾后重建与管理学院的博士研究生，方向是灾害护理。破坏性地震经常造成大量的人员伤亡和巨额经济损失，严重制约了社会的可持续发展。居民作为社区的重要组成部分，不仅直接经历地震灾害，而且在灾前预防以及灾后重建等方面都发挥着不可替代的作用。韧性理论强调系统对灾害环境的良好适应，并关注适应过程中各种保护性因素及其互动机制，对灾害实践有重要意义。本次访谈的主要目的是从护理视角出发，构建地震灾害背景下个体韧性量表，为减少灾害损失，促进灾后重建提供参考。现在是评价体系的构建阶段，需要您回答几个问题来协助我完善框架的构建，整个过程大概30分钟。在谈话的过程中您有任何的疑问或不适都请及时告诉我，如果您想中断或退出本次谈话都没有限制。我们的谈话内容将用录音笔录制下来，内容仅限于本次研究，我保证该资料绝对保密，在资料处理的每一步骤我都会保证您的身份不会泄露。您准备好，咱们现在可以开始了吗？

1. 您认为地震会给社区居民带来哪些影响？
2. 您认为地震会给社区居民带来哪些影响？
3. 您知道韧性吗，如果知道请问您从哪里了解到的韧性？
4. 您认为什么是个体韧性？
5. 如果从护理视角出发，您认为个体韧性都包括哪些方面呢？
6. 针对地震灾害，您上面提到的这些内容哪些是需要重点关注的？原因是什么，请详细地谈一下。
