# Supplementary material for: Developing a new individual earthquake resilience questionnaire: A reliability and validity test
Source: PLoS One. 2021 Jan 22;16(1):e0245662. doi: 10.1371/journal.pone.0245662 (PMC7822309; doi:10.1371/journal.pone.0245662)
Supplement: S2 File — (DOCX) [file pone.0245662.s002.docx]

**Informed consent of adolescent • Notification**

Dear participant/guardian:

Thank you very much for your participation in this study.

Earthquakes posed huge threats to many parts of the world. With urbanization accelerating, cities are more vulnerable to massive earthquakes. As an important part for community, residents not only directly experience the earthquake disaster, but also play an irreplaceable role in pre-disaster prevention and post-disaster reconstruction. The resilience theory emphasizes the system's good adaptation to adverse environment and pays attention to the protective factors and their interaction mechanism in the adaptation process, which is significant to disaster prevention and conrol. Adolescents are an important part of the community residents and it is important to understand their individual resilience which could improve the ability of residents for disaster reduction. The popuse of this study is to survey the resilience of adolescents in the background of the earthquake.

The study protocol was approved by the Human Subjects Ethics Sub-committee of Sichuan University (registration number: 2017-386). It will not pose any risk or harm to you or your family. The main research is to fill in the individual resilience questionnaire which focuses on your resilience in dealing with earthquake disasters. It will take about 5 minutes to complete. If you feel uncomfortable when fill in the questionnaire, you have the right to terminate the filling at any time.
 Your participation in this study is entirely voluntary. You may choose not to participate in the study, or withdraw from the study at any time, and you may not disclose the reason for your withdrawal, and your data will not be included in the study results.
 If you decide to participate in this study, your participation in this study will be confidential. We will not disclose any of your personal information to anyone without your consent. The research materials will only be used by the researcher to complete the study and will not be used for other purposes. When your data is used for research , we will use a number instead of your name. The results of this study will not reveal any personal information about you when published.
 At the end of the study, you will receive a set of stationery which is a small gift for you.
 Thanks again for your participation!

Thank you for reading the above information. If you have any questions about this study, please contact Ning Jiang at 13853803321.

**Informed consent • Consent signature page for participants**

Research Name: Construction of individual resilience questionnaire based on earthquake disaster from the perspective of nursing

**Research consent statement**

I have read this informed consent carefully and understood that this is a scientific study. The researcher has explained to me in detail the characteristics and possible problems of this study and given answers to relevant questions. I volunteered to participate in the study after fully understanding all the requirements for participants and the pros and cons of participating in the study. I fully understand:
 1. As a participant, I will comply with the instructions and requirements, voluntarily participate in this study, fully cooperate with the researchers, and provide relevant information to the researchers truthfully and objectively.
 2. The results of this study will only be used for scientific research purposes. My personal data will be kept confidential and protected in accordance with the law.
 3. My participation in this study was entirely voluntary. I understand that I can refuse to participate or withdraw from the study at any time without discrimination or retaliation, and my rights and interests will not be affected.

Phone: Phone:
Participant signature: Reseracher signature:
Guardian signature:

Date: Date:

**研究对象知情同意书** • **告知页**

尊敬的参与者/监护人：
您好！

破坏性地震经常造成大量的人员伤亡和巨额的经济损失，严重制约了社会的可持续发展。地震对世界许多地区都造成重大威胁，随着都市化进程的加快和城市发展占用更多的土地，导致城市更易于遭受重大地震影响而增大灾害的破坏性。社区居民作为社区的重要组成部分，不仅直接经历地震灾害，而且在灾前预防以及灾后重建等方面都发挥着不可替代的作用。韧性理论强调系统对灾害环境的良好适应，并关注适应过程中各种保护性因素及其互动机制，对灾害实践有重要意义。青少年作为社区居民的重要组成部分，了解其个体韧性十分重要。本研究的主要目的就是调查在地震背景下的青少年个体韧性，为提升社区韧性研究提供参考。

本实验方案已得到华西医院生物医学伦理分委会批准（registration number: 2017-386），遵循赫尔辛基宣言按照中国有关医学研究规范、法规进行。研究过程不会对您及您的家庭带来任何风险或伤害。主要的研究内容为问卷调查，需要您填写个体韧性调查问卷，问卷主要调查您在应对地震灾害时的韧性情况，完成本问卷大约需要花费5分钟的时间。在问卷填写时，若有心理不适，您有权随时终止填写。
 您参加本研究完全是自愿的。您可以选择不参加本研究，或在任何时候要求退出研究，并且可以不透露您的退出原因，您的数据将不纳入研究结果。
 如果您决定参加本项研究，您参加本研究的资料均属保密。在没有您同意的
前提下不向任何人公开您的任何个人信息，研究资料仅供本研究者完成论文使用，
不做其他用途。您的数据被用于研究分析时，我们会使用编号而不会用您的姓名。
这项研究结果发表时，不会暴露您个人的任何资料。
 在研究结束时，您会收到一套学习用品，这是我们送给您的小礼物。
 感谢您的参与！

感谢您阅读以上材料。如果您对本研究有任何的疑问，请拨电话13853803321与江宁联系。

**研究对象知情同意书 • 同意签字页**

研究项目名称：护理视角下基于地震灾害的个体韧性评价体系构建及实证研究

**研究同意申明**

我已仔细阅读本知情同意书，已了解这是一项科学研究，研究者已就此研究的特点和可能存在的问题向我做了详细解释，并对有关问题给予了解答。我在充分了解参与者须知的全部内容以及参加研究带来的利弊后，自愿参加本研究。我已充分理解：
 1.作为参与者，我将遵守参与者须知要求，自愿参加本研究，并与研究人员充分合作，如实、客观地向研究人员提供相关信息。
 2.本研究的结果只用于科研目的，我参加研究的个人资料均属保密，将依照法律规定得到保护。
 3.我参加本研究完全是自愿的。我明白可以拒绝参加或在任何时间退出研究，
而不会遭到歧视或报复，我的权益也不会因此受到影响。

联 系 电话： 联 系 电话：
参与者签名： 研究者签名：
监护人签名：
日 期： 年 月 日 日 期： 年 月 日
