# Supplementary material for: Developing a new individual earthquake resilience questionnaire: A reliability and validity test
Source: PLoS One. 2021 Jan 22;16(1):e0245662. doi: 10.1371/journal.pone.0245662 (PMC7822309; doi:10.1371/journal.pone.0245662)
Supplement: S1 Table — (DOCX) [file pone.0245662.s003.docx]

**S1 Table 1. Expert positive coefficient**

|  | **Number of sent questionnaires** | **Number of returned questionnaires** | **Response rate** |
| --- | --- | --- | --- |
| First round | 24 | 22 | 91.67% |
| Second round | 22 | 19 | 86.36% |

**S1 Table 2. Expert authority coefficient**

|  | **Cs** | **Ca** | **Cr** |
| --- | --- | --- | --- |
| First round | 0.83 | 0.91 | 0.87 |
| Second round | 0.82 | 0.89 | 0.86 |

**S1 Table 3. Expert coordination coefficient**

|  | **Number of items** | **Kendall’s W** | **P** |
| --- | --- | --- | --- |
| First round | 15 | 0.203 | <0.001 |
| Second round | 18 | 0.394 | <0.001 |
