# Supplementary material for: Developing a new individual earthquake resilience questionnaire: A reliability and validity test
Source: PLoS One. 2021 Jan 22;16(1):e0245662. doi: 10.1371/journal.pone.0245662 (PMC7822309; doi:10.1371/journal.pone.0245662)
Supplement: S2 Table — (DOCX) [file pone.0245662.s004.docx]

**Individual resilience questionnaire based on earthquake disaster from the perspective of nursing**

Dear Residents:

We are research team from the Institute for Disaster Management and Reconstruction, Sichuan University. Destructive earthquakes seriously restrict the sustainable development of society. As an integral part of the community, residents play an important role in disaster reduction, resistance and reconstruction. The main purpose of this study is to understand the resilience of the community residents in response to earthquake disasters and to provide a basis for improving the resilience of the community.

Please tick the box“√” that you think fits. This study is filled in anonymously. The answer has nothing to do with right or wrong, so please fill in the questionnaire according to your real situation. All contents you fill in will be strictly confidential and thank you very much for your cooperation.

**1. General situation of residents**

① Gender:

② Age:

③ Occupation:

④ Nationality:

⑤ Religious belief:

⑥ Education:

⑦ Registered permanent Residence:

⑧ Per capita annual income (ten thousand yuan) :

⑨ Time of residence in the community:

**2. Individual resilience questionnaire**

| Dimension | Item | Standard score | | | | |
| --- | --- | --- | --- | --- | --- | --- |
|  |  | Extremely match | Highly match | Basically match | Somewhat match | Extremely not match |
| Health status | I am generally in good health. |  |  |  |  |  |
|  | I can move freely and easily. |  |  |  |  |  |
|  | I can think clearly and communicate with others. |  |  |  |  |  |
| Mental resilience | I believe I can control my emotions when an earthquake strikes. |  |  |  |  |  |
|  | I always have a positive view when suffering difficulties. |  |  |  |  |  |
|  | I do not give up easily when suffering difficulties. |  |  |  |  |  |
|  | I can recover from setbacks quickly. |  |  |  |  |  |
|  | I believe that difficulties make me stronger. |  |  |  |  |  |
| Social adaptation | I can play my roles in daily life well, such as studying hard as a student, doing my own job well as a worker, taking care of my family as a parent, etc. |  |  |  |  |  |
|  | I can cope with problems well. |  |  |  |  |  |
|  | I can adapt quickly when the environment changes. |  |  |  |  |  |
|  | I can get along with others well. |  |  |  |  |  |
|  | I am good at finding and using social resource (staff, funds, supplies, skills, social relations and so on). |  |  |  |  |  |
| Disaster response capacity | I have basic earthquake disaster assessment ability.  Index illumination: I am aware of potential earthquake threats, can perceive earthquake warning signs, and preliminarily assess the grade of an earthquake and the risk of secondary disaster. |  |  |  |  |  |
|  | I know how to escape after shocks.  Index illumination: I know how to escape or avoid danger indoors and outdoors and self-rescue when trapped. |  |  |  |  |  |
|  | I have basic survival skills needed after an earthquake.  Index illumination: I have skills including getting water, food, and fuel and keeping water and food clean. |  |  |  |  |  |
|  | I can administer first aid.  Index illumination: I know the principles of searching for and rescuing those who are buried and have preliminary skills of first aid including hemostasis, bandages, CPR and carrying the wounded. |  |  |  |  |  |

**护理视角下基于地震灾害的个体韧性调查表**

尊敬的居民您好：

我是来自四川大学灾后重建与管理学院的调研小组。破坏性地震严重制约了社会的可持续发展。居民作为社区的组成部分是减灾、抗灾以及灾后重建的重要力量。本次调研的主要目的是了解社区居民地震灾害应对韧性，为提升社区的抗灾韧性提供依据。

请在您认为符合的一栏打“√”。本研究为匿名填写，答案无关对错，请按照您的真实情况填写即可。您填写的所有内容将被严格保密，请您放心填写。衷心感谢您的支持与配合！

**一、居民一般资料表**

（一）性别：

（二）年龄：

（三）职业：

（四）民族：

（五）宗教信仰：

（六）受教育程度：

（七）户口所在地：

（八）人均年收入（万元）：

（九）居住于本社区的时间：

二、**护理视角下基于地震灾害的个体韧性调查表**

| 维度 | 指标 | 评分标准 | | | | |
| --- | --- | --- | --- | --- | --- | --- |
|  |  | 完全符合 | 大部分符合 | 基本符合 | 少部分符合 | 完全不符合 |
| 健康状况 | 总的来说我的身体健康状况良好 |  |  |  |  |  |
|  | 我行动方便，活动自如 |  |  |  |  |  |
|  | 我思维清晰，能够与他人正常交流 |  |  |  |  |  |
| 心理韧性 | 地震发生时相信我能控制好自己的情绪 |  |  |  |  |  |
|  | 遇到困难时，我能够从积极的角度看待问题 |  |  |  |  |  |
|  | 面临困境时我不会轻易放弃 |  |  |  |  |  |
|  | 我能够从打击中较快走出，恢复信心 |  |  |  |  |  |
|  | 我相信经历磨难能让我变得更坚强 |  |  |  |  |  |
| 社会适应性 | 我能够胜任我在生活中担任的角色（如作为学生能够认真学习，作为职员能够做好本职工作，作为父母能够照顾好家庭等） |  |  |  |  |  |
|  | 我能够妥善处理遇到的问题 |  |  |  |  |  |
|  | 环境发生改变时我可以较快适应 |  |  |  |  |  |
|  | 我能够与周围的人和谐相处 |  |  |  |  |  |
|  | 我善于发现和使用社会资源（人力、物力、财力、技术以及社会关系等） |  |  |  |  |  |
| 地震灾害应对  能力 | 我具备地震灾害评估基本能力  指标说明：了解地震的危害及先兆，地震发生时能够初步判断其破坏程度和可能发生的次生灾害风险。 |  |  |  |  |  |
|  | 我了解避震逃生的方法  指标说明：了解室内外避险逃生方法及被困后自救方法。 |  |  |  |  |  |
|  | 我具备震后生存基本技能  指标说明：具备震后获取水源、食物、燃料以及安全用水、卫生饮食等基本技能。 |  |  |  |  |  |
|  | 我具备现场救护基本技能  指标说明：了解扒救被埋人员的原则，初步掌握伤口处理、徒手心肺复苏以及搬运伤员等现场救护方法。 |  |  |  |  |  |
